# Treatment Pathways and Candidate Correlates of Surgical Intervention in Locally Advanced Cervical and Other Gynecological Cancers: A Hypothesis-Generating Single-Center Cohort Study

**DOI:** 10.3390/jcm15186997

**Published:** 2026-09-10

**Authors:** Alexandru Orasan, Nicolae Constantin Balica, Adrian Mihail Sitaru, Mihaela Cristina Negru, Anda Ioana Morgovan, Kristine Guran, Andreea Mihaela Banta, Sebastian Ciurescu, Mihaela-Iuliana Sirbu, Eugen Horatiu Stefanescu

**Affiliations:** 1 Doctoral School in Medicine, “Victor Babes” University of Medicine and Pharmacy, 300041 Timisoara, Romania; alexandru.orasan@umft.ro (A.O.); anda.morgovan@umft.ro (A.I.M.); 2 Otorhinolaryngology (ENT) Department, “Victor Babes” University of Medicine and Pharmacy, Eftimie Murgu Square No. 2, 300041 Timisoara, Romania; balica@umft.ro (N.C.B.); mihaela.negru@umft.ro (M.C.N.); guran.kristine@umft.ro (K.G.); andreea.banta@umft.ro (A.M.B.); mihaela.sirbu@umft.ro (M.-I.S.); stefanescu@umft.ro (E.H.S.); 3 Department of Pediatric Surgery, “Louis Turcanu” Emergency Clinical Hospital for Children, Iosif Nemoianu Street 2, 300011 Timisoara, Romania

**Keywords:** locally advanced cervical cancer, gynecological cancers, induction chemotherapy, surgical salvage, hypothesis-generating study, geographic disparities, delta target dose, exploratory logistic regression, internal validation

## Abstract

**Background/Objectives**: Management of locally advanced cervical cancer (LACC) and other gynecological malignancies aims to achieve locoregional control while avoiding morbid surgical salvage. This exploratory, hypothesis-generating study described the treatment pathways of three protocol groups and examined whether clinical, sociodemographic, and treatment-deviation variables are associated with any oncological surgery. **Methods**: Sixty-nine adult patients with gynecological or urogenital cancers (89.9% cervical carcinoma) treated with platinum-based chemotherapy at a Romanian tertiary center between April 2024 and April 2025 were stratified into INTERLACE-type dose-dense induction chemotherapy followed by chemoradiotherapy (Group A, *n* = 27), classic three-weekly neoadjuvant chemotherapy followed by chemoradiotherapy (Group B, *n* = 7), and no induction chemotherapy (Group C, *n* = 35). Associations with any oncological surgery (upfront, completion, or salvage) were explored by multivariable logistic regression with bootstrap internal validation, Firth penalized regression, and restricted sensitivity analyses. **Results**: Twenty-five patients (36.2%) underwent surgery (14 upfront, 8 completion, 3 salvage), and none of them were from Group A, whose median follow-up was only 8.6 months and whose allocation was confounded by diagnosis and stage. The model showed an apparent area under the curve of 0.717 (optimism-corrected 0.672), accuracy of 73.9% against a no-information rate of 63.8% (*p* = 0.049), sensitivity of 0.480, and specificity of 0.886. Urban provenience (OR 0.343, 95% CI 0.113–1.039, *p* = 0.059), Delta Target Dose (OR 1.109 per Gy, *p* = 0.076), and number of chemotherapy cycles (OR 1.298 per cycle, *p* = 0.076) showed non-significant trends whose direction was unchanged in all sensitivity analyses. **Conclusions**: The absence of surgery after induction chemotherapy is hypothesis-generating rather than confirmatory, and the exploratory model is not a clinical prediction tool. Geographic provenience and planning-to-delivery treatment deviation are candidate variables for prospective evaluation in a homogeneous LACC population with standardized radiotherapy parameters and mature oncological endpoints.

## 1. Introduction

Cervical cancer remains the fourth most frequent cancer in women worldwide, with an estimated 660,000 new cases and 350,000 deaths in 2022 [1], and its burden is concentrated in regions where screening coverage is low and a large proportion of patients present with locally advanced disease, as is the case in Romania [2,3]. The global conceptual framework for managing locally advanced cervical cancer (LACC) and other locally advanced gynecological malignancies rests on definitive concurrent platinum-based chemoradiotherapy (CCRT) with image-guided brachytherapy, which has been the standard of care since the demonstration that concurrent cisplatin improves survival over radiotherapy alone [4,5,6,7,8]. Image-guided adaptive brachytherapy has since further improved pelvic control and survival in large multicenter cohorts [9,10]. In this paradigm, chemoradiotherapy is itself the definitive treatment, and surgery is reserved for three clinically distinct situations: planned upfront surgery in early-stage or selected non-cervical disease, where fertility-sparing options may also be considered [11], completion surgery for histologically or radiologically confirmed residual disease after chemoradiotherapy, and salvage surgery for locoregional recurrence [4,12,13]. Completion and salvage procedures in a previously irradiated pelvis, up to and including pelvic exenteration, carry substantial morbidity, so that avoiding them without compromising local control is a central objective of multidisciplinary management [12,13,14,15,16].

Chemotherapy treatment intensity (CTI), understood here as the schedule, dose density, and number of platinum-containing cycles delivered before and during radiotherapy, has re-emerged as a modifiable determinant of outcome. The number of concurrent cisplatin cycles is independently associated with survival after CCRT [17], and the interaction between the number of cycles and brachytherapy dose–volume parameters influences local control [18]. Most importantly, the GCIG INTERLACE phase 3 trial showed that six weekly cycles of dose-dense carboplatin and paclitaxel given immediately before standard CCRT improve progression-free and overall survival in LACC [19,20], whereas conventional three-weekly neoadjuvant chemotherapy followed by radical surgery did not outperform CCRT in two randomized comparisons [21,22]. More recently, the addition of pembrolizumab to CCRT improved overall survival in high-risk LACC [23], confirming that intensification of the definitive non-surgical pathway, rather than a return to radical surgery, is the direction of current standard-setting trials. The INTERLACE regimen was adopted by our tumor board in 2024 for eligible patients with LACC; the two other pathways in use during the study period were a classic three-weekly neoadjuvant platinum–taxane protocol followed by CCRT and a no-induction pathway consisting of upfront surgery or definitive CCRT [4,19,21].

Whether patients ultimately require surgery after these pathways is also shaped by factors outside the tumor. In Romania, cervical cancer mortality remains among the highest in Europe and displays marked rural–urban inequalities [24], and geography has been proposed as an independent prognostic and decision-making factor [25,26]. Physical treatment quality is a further candidate: the dose actually delivered to the target may deviate from the dose prescribed at planning because of anatomical change, adaptive replanning, treatment interruptions, or incomplete brachytherapy, and interfraction dose variations have been linked to clinical outcome [27,28]. Within the EMBRACE framework, tumor dose, target volume, and overall treatment time are established determinants of local control after chemoradiotherapy with brachytherapy [29], so that deviations between planned and delivered treatment are a legitimate object of study even when they are measured coarsely.

The present study is explicitly exploratory and hypothesis-generating. Its objectives were (i) to describe the treatment pathways and surgical outcomes of three protocol groups treated at a single Romanian tertiary center during the first year of INTERLACE-type induction chemotherapy; (ii) to characterize the radiotherapy component of treatment; and (iii) to explore, in a multivariable model with internal validation and pre-specified sensitivity analyses, whether geographic provenience, age, planning-to-delivery treatment deviation (Delta Target Dose, an exploratory institutional treatment-deviation metric), and chemotherapy intensity (an exploratory correlate) are associated with the need for any oncological surgery. Given the sample size, the heterogeneity of the cohort, and the short follow-up, the study does not aim to deliver a clinical prediction tool but to identify candidate variables for a subsequent prospective study in a homogeneous LACC population.

## 2. Materials and Methods

### 2.1. Study Design and Setting

We conducted a single-center, observational, hypothesis-generating cohort study at a tertiary oncology center in Timișoara, Romania. Consecutive adult patients with a histologically confirmed gynecological or urogenital malignancy who started platinum-based chemotherapy between 1 April 2024 and 1 April 2025 were enrolled prospectively in an institutional registry, with a data lock on 1 April 2025. The study was conducted in accordance with the Declaration of Helsinki and its later amendments and was approved by the Institutional Review Board of the Victor Babes University of Medicine and Pharmacy Timișoara (approval no. 30, dated 19 April 2024) before the first patient was enrolled. All patients gave written informed consent for treatment, for inclusion in the institutional registry, and for the use of their pseudonymized clinical, dosimetric, and imaging data for research purposes; consent could be withdrawn at any time without consequences for care. Data were pseudonymized at extraction, stored on a password-protected institutional server in accordance with the General Data Protection Regulation (EU 2016/679), and accessed only by the investigators named in the protocol. No patient-identifying information is reported. Reporting follows the TRIPOD recommendations for exploratory prediction research [30].

### 2.2. Participants and Treatment Groups

**Figure 1:**
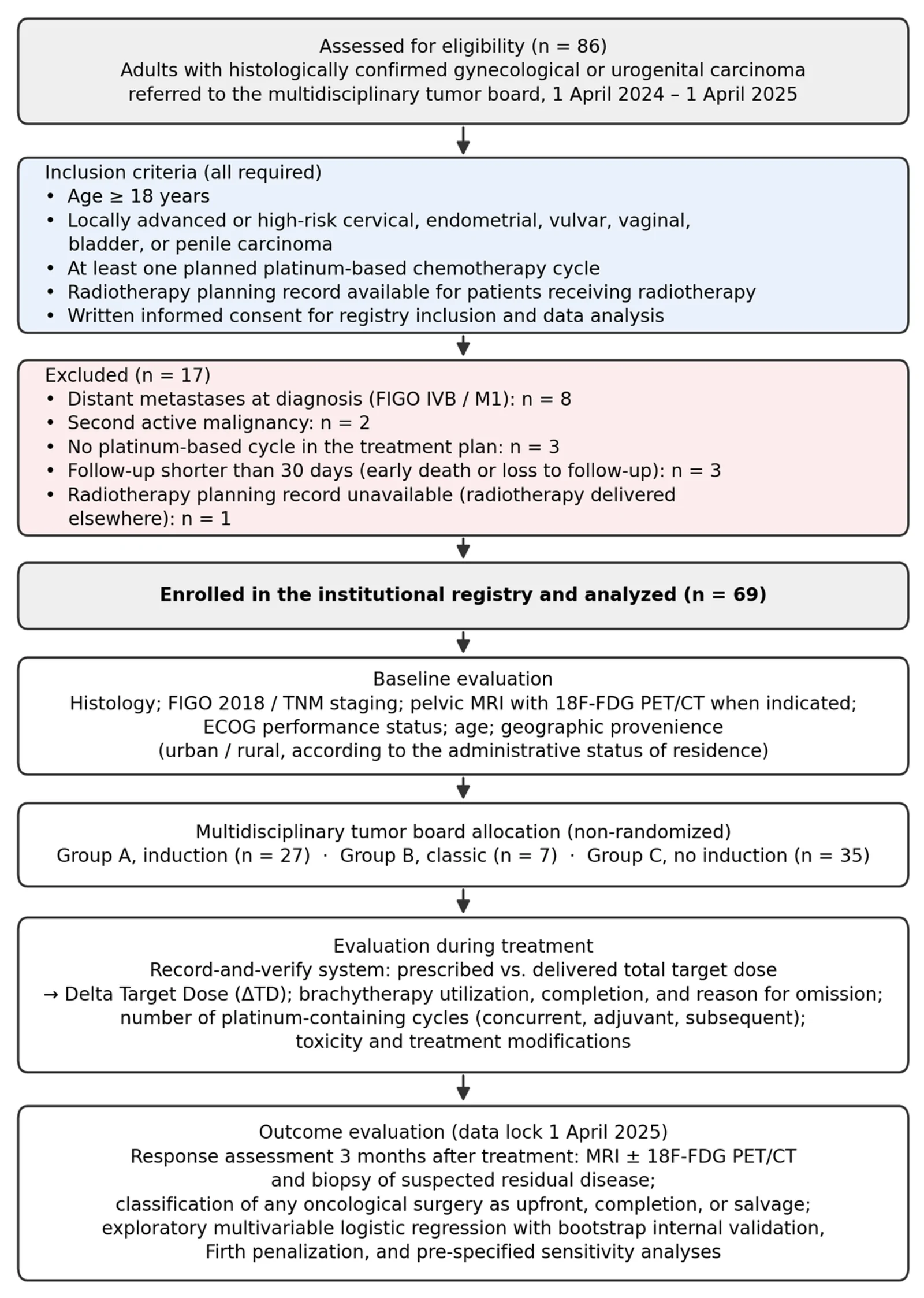
Flow diagram of patient selection and evaluation. The inclusion and exclusion criteria, the number of patients excluded for each reason, and the evaluation methods applied at baseline, during treatment, and at outcome assessment are shown. Arrows indicate the sequence of the selection and evaluation steps; the blue-shaded box lists the inclusion criteria and the red-shaded box the exclusion criteria. CCRT, concurrent platinum-based chemoradiotherapy; ECOG, Eastern Cooperative Oncology Group; FIGO, International Federation of Gynecology and Obstetrics; MRI, magnetic resonance imaging; 18F-FDG PET/CT, 18F-fluorodeoxyglucose positron emission tomography/computed tomography; ΔTD, Delta Target Dose.

**Figure 2:**
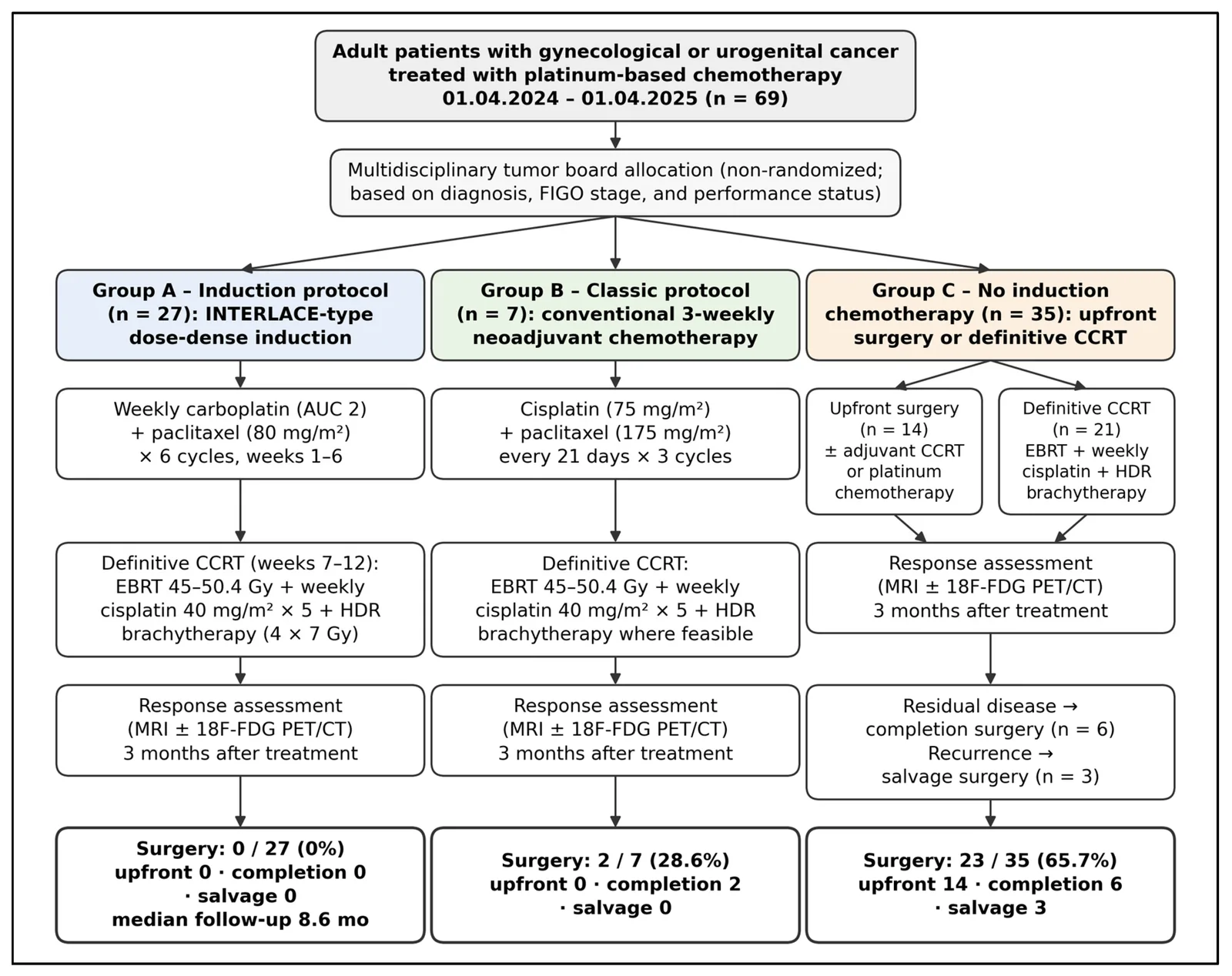
Treatment pathways of the three protocol groups and surgical outcomes by the data lock. Group A, INTERLACE-type dose-dense induction chemotherapy followed by definitive chemoradiotherapy; Group B, classic three-weekly neoadjuvant chemotherapy followed by chemoradiotherapy; Group C, no induction chemotherapy (upfront surgery or definitive chemoradiotherapy). CCRT, concurrent platinum-based chemoradiotherapy; EBRT, external beam radiotherapy; HDR, high-dose-rate brachytherapy.

Inclusion criteria were: (i) age ≥ 18 years; (ii) histologically confirmed carcinoma of the uterine cervix (FIGO 2018 stage IB3–IVA) or locally advanced or high-risk carcinoma of the endometrium, vulva, vagina, urinary bladder, or penis, for which the multidisciplinary tumor board recommended curative-intent multimodal treatment; (iii) a treatment plan containing at least one platinum-based chemotherapy cycle, whether as induction, concurrent, or adjuvant treatment; (iv) availability of the radiotherapy planning and record-and-verify record for every patient who received radiotherapy, so that the Delta Target Dose could be computed; and (v) written informed consent. Exclusion criteria were: (i) distant metastases at diagnosis (FIGO IVB or M1 disease); (ii) a second active malignancy; (iii) fewer than 30 days of follow-up after the start of treatment because of early death or loss to follow-up; and (iv) delivery of radiotherapy at another institution without an available planning record. Of 86 patients assessed for eligibility, 17 were excluded (distant metastases, *n* = 8; second active malignancy, *n* = 2; no platinum-based cycle in the treatment plan, *n* = 3; follow-up shorter than 30 days, *n* = 3; unavailable radiotherapy planning record, *n* = 1), leaving 69 patients for analysis. The selection process and the evaluation methods applied at baseline, during treatment, and at outcome assessment are summarized in Figure 1. Treatment allocation was decided by the multidisciplinary tumor board according to diagnosis, FIGO/TNM stage, performance status, and patient preference and was not randomized. Three mutually exclusive treatment groups were defined a priori (Figure 2):

Group A, INTERLACE-type induction protocol (*n* = 27): six weekly cycles of carboplatin (AUC 2) and paclitaxel (80 mg/m^2^) followed within 7 days by definitive CCRT, as in the GCIG INTERLACE trial [19]. Only patients with cervical carcinoma FIGO IIB–IVA were eligible.
Group B, classic protocol (*n* = 7): conventional neoadjuvant chemotherapy with cisplatin (75 mg/m^2^) and paclitaxel (175 mg/m^2^) every 21 days for three cycles, followed by definitive CCRT.
Group C, no induction chemotherapy (*n* = 35): either planned upfront surgery followed by adjuvant platinum-based chemotherapy with or without external beam radiotherapy (*n* = 14), or definitive CCRT without preceding chemotherapy (*n* = 21).

### 2.3. Radiotherapy and Brachytherapy

All radiotherapy was delivered with volumetric modulated arc therapy after CT simulation with MRI co-registration, following ESGO/ESTRO/ESP and EMBRACE II planning aims [31,32]. External beam radiotherapy (EBRT) to the pelvis (± para-aortic region) was prescribed at 45 Gy in 25 fractions or 50.4 Gy in 28 fractions; radiologically involved nodes received a simultaneous integrated boost (SIB) to 55–57.5 Gy in 25 fractions. Concurrent cisplatin 40 mg/m^2^ was given weekly for five planned cycles, with carboplatin AUC 2 substituted when creatinine clearance was <50 mL/min. Image-guided high-dose-rate (HDR) brachytherapy (CT- or MRI-based, intracavitary ± interstitial) was planned as 4 × 7 Gy to the high-risk clinical target volume (HR-CTV), with a departmental planning aim of a cumulative EQD2 D90 ≥ 85 Gy to the HR-CTV and D2cc constraints of ≤90 Gy (bladder) and ≤75 Gy (rectum, sigmoid); when brachytherapy was anatomically unfeasible or declined, an EBRT boost was delivered instead. The intended overall treatment time (OTT) was ≤56 days. Brachytherapy utilization, completion, and the reason for omission were recorded for every patient treated with definitive intent. Individual HR-CTV D90 values, organ-at-risk doses, OTT, and treatment interruptions were not extracted into the study database and could therefore not be analyzed; this is addressed in the limitations.

### 2.4. Outcome and Candidate Variables

The outcome was any oncological surgery performed by the data lock, coded as a binary variable and further classified by indication as planned upfront surgery, completion surgery for residual disease at the 3-month post-treatment assessment (MRI, with 18F-FDG PET/CT when equivocal, and biopsy confirmation), or salvage surgery for locoregional recurrence. We acknowledge that these are clinically distinct events; the composite was used because the number of post-treatment surgeries alone (*n* = 11) was too small for multivariable modeling, and a sensitivity analysis restricted to post-treatment surgery is reported (Section 2.6).

Four candidate variables were pre-specified on clinical grounds and to respect the events-per-variable constraint [33,34]: geographic provenience (urban vs. rural, according to the administrative status of the patient’s residence), age at diagnosis (years), the Delta Target Dose (ΔTD), and chemotherapy intensity expressed as the number of platinum-containing cycles. The ΔTD is an exploratory institutional treatment-deviation metric rather than an established dosimetric endpoint or a conventional dosimetric predictor. It was defined as the absolute difference, in Gy of physical dose, between the total dose to the primary target (EBRT plus brachytherapy or EBRT boost) prescribed at initial planning and the total dose actually delivered, as documented in the record-and-verify system at the end of treatment. A ΔTD of 0 Gy therefore indicates that the plan was delivered as prescribed, whereas positive values arise from adaptive replanning or additional boost for poor response or anatomical change, from replacement of brachytherapy by an EBRT boost, or from dose reduction or premature termination. Because the ΔTD is an absolute (unsigned) difference, clinically opposite situations, namely additional dose or boosting on the one hand and dose reduction or premature termination on the other, generate similar positive values despite representing very different treatment courses; the metric therefore records that a deviation occurred and its magnitude, but not its direction or its clinical meaning, and it was analyzed only as an exploratory correlate of the surgical pathway, not as a dosimetric predictor. Patients who received no radiotherapy (*n* = 11) were assigned a ΔTD of 0 Gy in the primary analysis; because this convention conflates absence of radiotherapy with exact delivery, a sensitivity analysis restricted to irradiated patients was pre-specified (Section 2.6). The number of chemotherapy cycles counted all concurrent, adjuvant, or subsequent platinum-containing cycles delivered by the data lock; induction cycles were not counted, because they define Group A and would otherwise duplicate the protocol variable. Because this count aggregates cycles delivered at different stages of the clinical course (concurrent, adjuvant, and subsequent), some of them after the decision to operate, it was treated throughout as an exploratory correlate (candidate variable) of the surgical pathway rather than as a predictor.

### 2.5. Statistical Analysis

Continuous variables are summarized as mean ± standard deviation (SD) or median (interquartile range, IQR) and compared with Student’s t-test, one-way analysis of variance (ANOVA), or the Mann–Whitney U test according to distribution; categorical variables are given as counts (%) and compared with Fisher’s exact test. An exploratory one-way ANOVA compared the ΔTD across primary diagnoses, with and without the two urogenital cases; only the test statistics are reported, because several diagnostic strata contained fewer than three patients. Binary logistic regression was chosen because the outcome is dichotomous, the number of events (25) allowed a maximum of four covariates at approximately six events per variable [33,34], and the odds ratio (OR) is directly interpretable for tumor board discussion. The treatment group could not be entered as a covariate because no surgery occurred in Group A (perfect separation); the association between group and surgery is therefore reported descriptively. Multicollinearity was checked with variance inflation factors (VIF), calibration with the Hosmer–Lemeshow test and calibration slope, and discrimination with the area under the receiver operating characteristic curve (AUC). Internal validation used 1000 bootstrap resamples to estimate optimism-corrected AUC and calibration slope. Classification metrics (accuracy, sensitivity, specificity, predictive values) were computed at a probability threshold of 0.5, and accuracy was compared with the no-information rate (NIR, the proportion of the majority class) by an exact binomial test. Because maximum-likelihood estimates are biased away from the null in small samples, Firth’s penalized likelihood logistic regression was fitted as an alternative modeling approach [35]. Two-sided *p* < 0.05 was considered statistically significant; no adjustment for multiplicity was made because the analysis is exploratory. Analyses were performed in R version 4.4 (packages rms, logistf, pROC).

### 2.6. Pre-Specified Sensitivity Analyses

Four sensitivity analyses addressed the main structural concerns of the design: (a) the primary model refitted with Firth penalization; (b) the primary model restricted to the 58 irradiated patients, in whom the ΔTD is a true planning-to-delivery deviation; (c) the primary model restricted to the 62 patients with cervical carcinoma; and (d) the outcome redefined as post-treatment surgery only (completion or salvage), after exclusion of the 14 patients with planned upfront surgery. Because analysis (d) contained only 11 events, it was limited to univariable comparisons.

## 3. Results

### 3.1. Cohort Characteristics

Of 86 patients assessed for eligibility, 17 were excluded and 69 were analyzed (Figure 1); their baseline characteristics are given in Table 1. Mean age was 54.3 ± 11.2 years (range 29–79), 67 patients (97.1%) were women, and 31 (44.9%) lived in rural areas. Cervical carcinoma accounted for 62 cases (89.9%; 59 squamous, 3 adenocarcinoma), with endometrial (*n* = 2), vulvar (*n* = 2), vaginal (*n* = 1), and urogenital carcinoma (*n* = 2; one urothelial bladder carcinoma and one penile squamous carcinoma) making up the remainder. Group A contained only cervical carcinoma FIGO IIB–IVA, whereas all non-cervical diagnoses and all FIGO IB3–IIA cervical tumors were in Group C. Median follow-up from the start of treatment was 11.2 months (IQR 7.4–14.9) overall and 8.6 months (IQR 5.9–10.8) in Group A.

### 3.2. Radiotherapy and Brachytherapy Characteristics

Fifty-eight patients (84.1%) received radiotherapy: 53 with definitive intent (all of Groups A and B and 19 of the 21 Group C patients planned for definitive CCRT; two progressed before radiotherapy and received palliative platinum-based chemotherapy) and 5 as adjuvant EBRT after upfront surgery. The 11 non-irradiated patients were 9 patients treated with upfront surgery and adjuvant chemotherapy only and the 2 patients who progressed before radiotherapy. Radiotherapy characteristics are summarized in Table 2. Brachytherapy was delivered in 41 of the 53 definitive-intent patients (77.4%) and completed as planned in 36 (87.8% of those started); it was replaced by an EBRT boost in 5 patients, declined or contraindicated in 4, and precluded by progression in 3. Brachytherapy utilization did not differ significantly between groups (*p* = 0.61). Among irradiated patients the ΔTD was 0 Gy in 17 (29.3%), median 1.8 Gy (IQR 0–4.2), and ≥ 5 Gy in 12 (20.7%); the main sources of deviation were adaptive replanning or additional boost (*n* = 14), replacement of brachytherapy by an EBRT boost (*n* = 5), and dose reduction or premature termination (*n* = 9). In an exploratory one-way ANOVA, the ΔTD differed across primary diagnoses (F(5,63) = 3.32, *p* = 0.010); after exclusion of the two urogenital cases the difference was not statistically significant (F(4,60) = 1.29, *p* = 0.284).

### 3.3. Surgical Outcomes by Type and Treatment Group

Twenty-five patients (36.2%) underwent oncological surgery by the data lock (Table 3). Fourteen procedures were planned upfront operations (radical hysterectomy with pelvic lymphadenectomy in 8 cervical cases, total hysterectomy with bilateral salpingo-oophorectomy in 2 endometrial cases, radical vulvectomy in 2, radical cystectomy in 1, partial penectomy in 1); 8 were completion procedures for biopsy-confirmed residual cervical disease at the 3-month assessment (extrafascial hysterectomy in 5, radical hysterectomy in 3); and 3 were salvage procedures for central recurrence (pelvic exenteration in 2, radical hysterectomy in 1). No patient in Group A had undergone surgery, compared with 2 of 7 (28.6%) in Group B and 23 of 35 (65.7%) in Group C (Fisher *p* < 0.001); after exclusion of the 14 planned upfront procedures, which by design belong to Group C, post-treatment surgery occurred in 0 of 27, 2 of 7, and 9 of 21 patients, respectively (*p* < 0.001). The median interval from the end of primary treatment to completion or salvage surgery was 4.1 months (range 1.8–9.6). Surgery was more frequent in rural (15/31, 48.4%) than in urban patients (10/38, 26.3%; crude OR 0.38 for urban residence, 95% CI 0.14–1.04, *p* = 0.079).

### 3.4. Exploratory Multivariable Analysis

**Figure 3:**
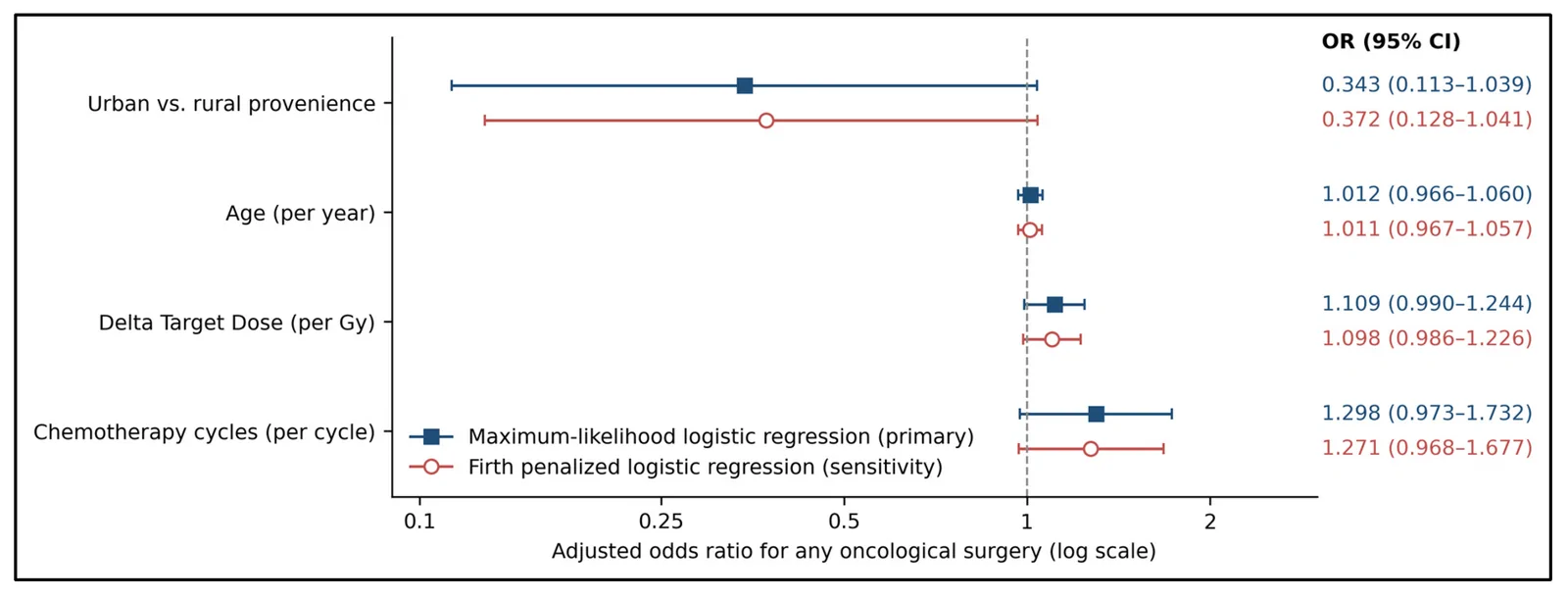
Forest plot of adjusted odds ratios for any oncological surgery in the primary maximum-likelihood model and in the Firth penalized sensitivity model (log scale; error bars are 95% confidence intervals). The vertical dashed line marks an odds ratio of 1 (no association). No covariate reached statistical significance.

**Figure 4:**
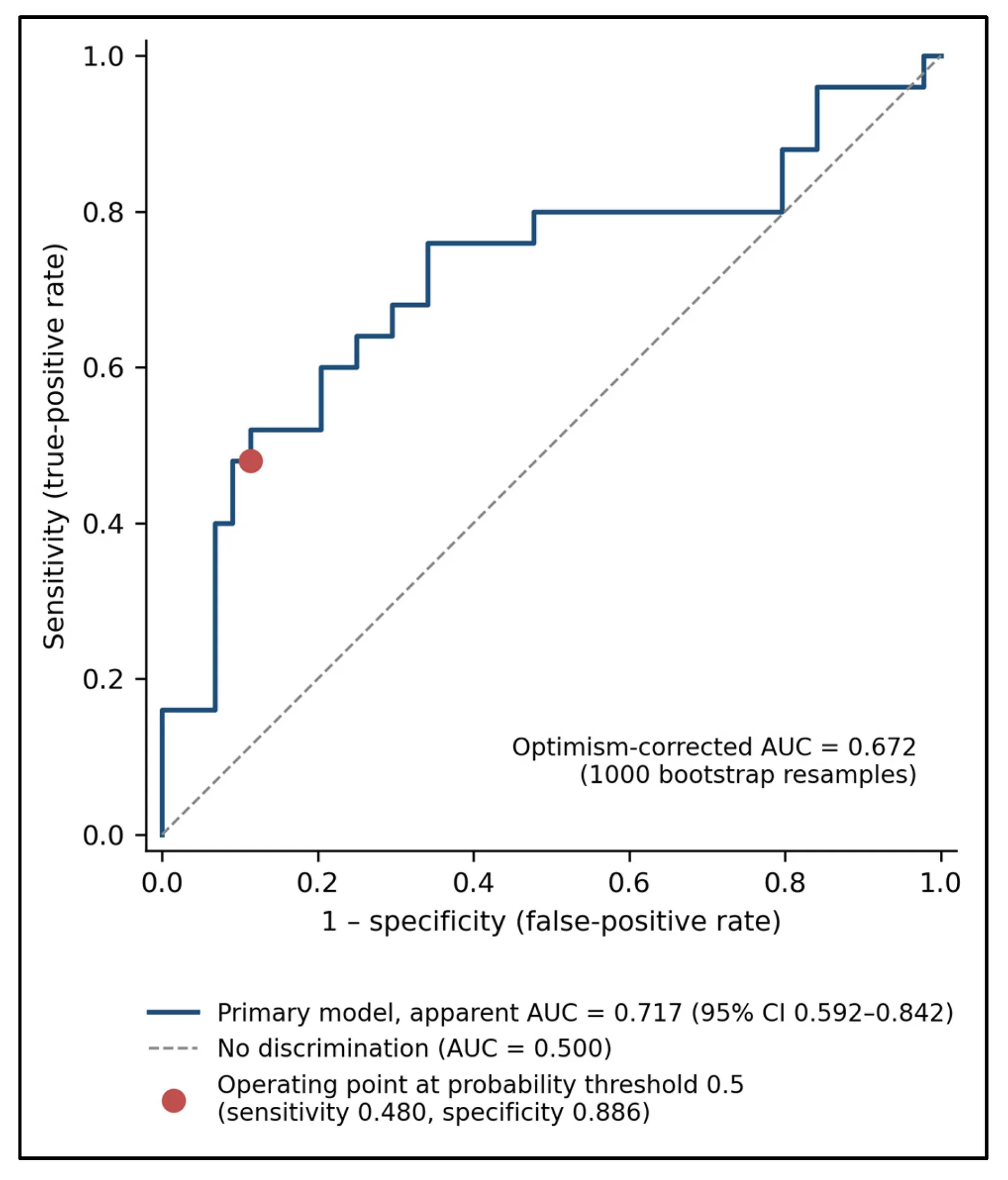
Receiver operating characteristic (ROC) curve of the primary exploratory logistic regression model for any oncological surgery (*n* = 69, 25 events). The apparent area under the curve (AUC) was 0.717 (95% CI 0.592–0.842) and the optimism-corrected AUC after 1000 bootstrap resamples was 0.672. The marked point is the operating point at the 0.5 probability threshold (sensitivity 0.480, specificity 0.886); the dashed diagonal indicates no discrimination.

In univariable analysis, urban provenience (crude OR 0.38, 95% CI 0.14–1.04, *p* = 0.079), age (OR 1.008 per year, 95% CI 0.966–1.052, *p* = 0.72), ΔTD (OR 1.12 per Gy, 95% CI 1.00–1.25, *p* = 0.052), and number of chemotherapy cycles (OR 1.27 per cycle, 95% CI 0.97–1.66, *p* = 0.081) were associated with surgery in the same direction as in the adjusted model, and none reached statistical significance. The primary logistic regression model (Table 4, Figure 3) included provenience, age, ΔTD, and number of chemotherapy cycles (25 events, 6.25 events per variable; all VIF ≤ 1.21). Urban provenience was associated with lower odds of surgery (OR 0.343, 95% CI 0.113–1.039, *p* = 0.059), and higher ΔTD (OR 1.109 per Gy, 95% CI 0.990–1.244, *p* = 0.076) and more chemotherapy cycles (OR 1.298 per cycle, 95% CI 0.973–1.732, *p* = 0.076) showed trends toward higher odds; age was not associated (OR 1.012 per year, 95% CI 0.966–1.060, *p* = 0.61). No covariate reached statistical significance. Nagelkerke R^2^ was 0.187 and the Hosmer–Lemeshow test indicated no gross miscalibration (χ^2^ = 6.9, 8 df, *p* = 0.55). The apparent AUC was 0.717 (95% CI 0.592–0.842; Figure 4) and decreased to 0.672 after bootstrap optimism correction, with a calibration slope of 0.781. At a 0.5 threshold the model classified 51 of 69 patients correctly (accuracy 73.9%, 95% CI 61.9–83.7) against an NIR of 63.8% (exact binomial *p* = 0.049), with sensitivity 0.480 (12/25), specificity 0.886 (39/44), positive predictive value 0.706, negative predictive value 0.750, and Cohen’s κ 0.39; that is, 13 of the 25 patients who underwent surgery were not identified. The full classification table is given in Table 5.

### 3.5. Sensitivity Analyses

Firth penalization attenuated all coefficients slightly toward the null without changing their direction (Table 4, Figure 3). In the 58 irradiated patients, in whom the ΔTD represents a true planning-to-delivery deviation, the estimate for ΔTD was essentially unchanged (OR 1.097 per Gy, 95% CI 0.968–1.243, *p* = 0.15) and the provenience estimate remained of the same magnitude (OR 0.31, *p* = 0.066). Restriction to the 62 cervical carcinoma patients likewise reproduced the direction and magnitude of all estimates, with wider confidence intervals. An alternative specification adding an indicator for receipt of radiotherapy to the primary model was not estimable, because 9 of the 11 non-irradiated patients had undergone upfront surgery and the indicator was nearly collinear with the outcome pathway. When the outcome was restricted to post-treatment surgery (11 events among the 55 patients without upfront surgery), rural patients underwent completion or salvage surgery more often than urban patients (7/23, 30.4% vs. 4/32, 12.5%; Fisher *p* = 0.17), patients who required post-treatment surgery had a higher ΔTD (median 3.1 vs. 0.8 Gy, Mann–Whitney *p* = 0.06) and had received more chemotherapy cycles (5.6 ± 1.8 vs. 4.3 ± 1.7, *p* = 0.04). Optimism-corrected discrimination was ≤0.675 in every model.

## 4. Discussion

In this single-center cohort treated during the first year of INTERLACE-type induction chemotherapy, 36.2% of patients underwent oncological surgery, of which more than half were planned upfront procedures and only 11 were completion or salvage operations after chemoradiotherapy. No patient in the induction group required surgery at a median follow-up of 8.6 months. Rural provenience, a higher planning-to-delivery dosimetric deviation, and a larger number of chemotherapy cycles were associated with surgery only as non-significant trends, and these trends were reproduced in direction across Firth-penalized, irradiated-only, cervical-only, and post-treatment-surgery analyses. The exploratory model had fair apparent but modest optimism-corrected discrimination and identified fewer than half of the patients who underwent surgery. The findings are therefore hypothesis-generating: they describe contemporary real-world pathways and nominate candidate variables, but they do not establish predictors and do not constitute a clinical prediction tool.

The observation that none of the 27 patients treated with dose-dense induction chemotherapy underwent surgery must be interpreted with caution. Definitive CCRT with image-guided brachytherapy is itself the curative treatment for LACC [4,5,27], so the absence of subsequent surgery is not an established surrogate of treatment efficacy; in a pathway that never intends surgery, surgery-free status mainly reflects the absence of documented residual or recurrent disease within the observation window, whereas the risk of recurrence in LACC extends well beyond the first year [36]. Three features of our design further restrict inference. Allocation was non-randomized and confounded by diagnosis and stage, because Group A consisted exclusively of cervical carcinoma FIGO IIB–IVA whereas all upfront surgical candidates were in Group C. Follow-up in Group A (8.6 months) was shorter than the longest observed interval to completion or salvage surgery in the rest of the cohort (9.6 months), so that a proportion of induction patients had not yet passed the point at which such a decision would be made. Finally, perfect separation prevented inclusion of the treatment group in the regression, and the comparison between groups is purely descriptive. The INTERLACE trial demonstrated a survival benefit of induction chemotherapy with mature follow-up and clinically meaningful endpoints [19]; our data are consistent with the feasibility of that pathway in a Romanian tertiary center but cannot address its efficacy or its effect on the need for surgery.

Rural patients underwent surgery more often than urban patients (48.4% vs. 26.3%), an association that persisted in adjusted analysis (OR 0.343 for urban residence, 95% CI 0.113–1.039, *p* = 0.059) and in each sensitivity analysis, but that did not reach conventional statistical significance. Rural–urban inequalities in cervical cancer incidence and mortality are well documented in Romania and elsewhere [24,25,26]. A plausible mechanism links rural residence to travel burden, treatment interruptions, and prolonged OTT, because extending OTT beyond approximately 55 days permits accelerated repopulation and reduces radiotherapy efficacy [37]. We emphasize, however, that none of the intermediate steps of this pathway (travel distance, OTT, number and duration of interruptions, adherence) was measured in the present study, and that part of the association may simply reflect the higher proportion of rural patients presenting with stages for which upfront surgery was chosen. Provenience should therefore be regarded strictly as an exploratory marker whose mechanism is untested; prospective collection of OTT, travel distance, and interruption data is planned in our next cohort to test it.

Beyond sociodemographic factors, physical and systemic treatment intensity are candidate correlates of the surgical pathway. We operationalized them as the Delta Target Dose (ΔTD, in Gy) and the number of platinum-containing chemotherapy cycles, as defined in Section 2.4, and entered both into the exploratory regression model. The rationale, derivation, and interpretation of each are considered in turn.

Modern radiotherapy relies on complex planning to calculate doses covering high-risk clinical target volumes (HR-CTV) [27]. Because physical delivery involves anatomical variations, the Delta Target Dose quantifies the variance between planned prescriptions and the actual delivery required for adequate target coverage. The ΔTD is not a conventional clinical or dosimetric endpoint or predictor; we introduced it as an exploratory institutional treatment-deviation metric. Conceptually it captures three distinct phenomena that all signal a departure from the intended treatment: (i) adaptive replanning or additional boost required because of tumor regression or persistence and anatomical change during treatment, (ii) replacement of brachytherapy by an EBRT boost when the applicator geometry became unfeasible, and (iii) dose reduction or premature termination for toxicity or non-adherence. In each case a larger deviation indicates that the treatment actually received differed from the treatment planned, which is biologically plausible as a correlate of incomplete response. An important limitation follows directly from this definition: because the ΔTD is an absolute difference, additional dose or boosting and dose reduction or premature termination, which are clinically opposite situations, produce similar positive values, so that a given ΔTD value cannot be traced back to the treatment course that generated it. Interfraction dose variations in target and organs at risk have been associated with clinical outcome in HDR brachytherapy [28], and the ΔTD can be regarded as a coarse, whole-course analogue of such variation. It does not, however, replace the established EMBRACE-type parameters (HR-CTV D90, D2cc, OTT) [31], which were not available in our database.

In our model, the Delta Target Dose yielded an odds ratio of 1.109 per Gy (95% CI 0.990–1.244, *p* = 0.076), a non-significant trend linking incremental dosimetric variance to a higher likelihood of surgery. An exploratory ANOVA (Section 3.2) suggested that this metric varies with primary diagnosis (F(5,63) = 3.32, *p* = 0.010), but that result was generated entirely by the two urogenital carcinoma cases and disappeared when they were excluded (F(4,60) = 1.29, *p* = 0.284); it should not be interpreted as a diagnosis-dependent dosimetric effect. Two further caveats apply. First, assigning 0 Gy to the 11 non-irradiated patients conflates the absence of radiotherapy with exact delivery; the pre-specified analysis restricted to irradiated patients produced an essentially identical estimate (OR 1.097 per Gy, *p* = 0.15), which suggests that this coding did not drive the trend, although with 16 events it cannot exclude a structural bias. Second, because the ΔTD increases both when a poorly responding tumor is boosted and when treatment is curtailed, it is partly a consequence of the same clinical course that leads to surgery, and the association should be read as a correlation within the treatment pathway rather than as a causal predictor.

The number of chemotherapy cycles, an exploratory correlate that aggregates concurrent, adjuvant, and subsequent cycles delivered at different stages of the clinical course, showed a non-significant trend toward higher odds of surgery (OR 1.298 per cycle, 95% CI 0.973–1.732, *p* = 0.076), and patients who required post-treatment surgery had received more cycles (5.6 vs. 4.3, *p* = 0.04). At first sight this is paradoxical, because the number of concurrent cisplatin cycles is associated with better survival after CCRT [17] and interacts favorably with brachytherapy dose–volume parameters [18]. Three explanations, none of which implies that chemotherapy is harmful, are more plausible. First, the variable counted all platinum-containing cycles delivered by the data lock, including adjuvant cycles after upfront surgery and additional cycles administered for residual or recurrent disease before completion or salvage surgery; the count is therefore partly a consequence of the clinical course that leads to surgery (reverse causation, a form of confounding by indication). Second, the induction cycles of Group A, in which no surgery occurred, were deliberately excluded from the count to avoid duplicating the protocol variable, so the variable does not capture the one form of chemotherapy intensification for which a benefit has been demonstrated [19]. Third, with 25 events the estimate is imprecise and the confidence interval includes an inverse association. The direction of the trend was unchanged in the Firth, irradiated-only, and cervical-only analyses, which argues against an artifact of small-sample bias but not against confounding by indication. For all these reasons, the chemotherapy-cycle variable is presented as an exploratory correlate, or candidate variable for future study, and not as a predictor of surgery. A prospective study should therefore record cycles by phase (induction, concurrent, adjuvant, subsequent) and time-stamp them relative to the surgical decision. Furthermore, “surgical requirement” was treated as a binary outcome, failing to granularly differentiate between minor extrafascial hysterectomies and highly complex total pelvic exenterations. Given their drastically different morbidity profiles, future studies with an adequate number of events should model surgery type (upfront, completion, salvage) and extent separately, for example, with multinomial regression.

An apparent AUC of 0.717 that falls to 0.672 after optimism correction, with a calibration slope of 0.781, sensitivity of 0.480, and an accuracy only marginally above the NIR, characterizes a model with limited discriminatory ability and evident overfitting. This is in line with what can be expected from clinical and sociodemographic variables alone: models that predict residual disease or treatment response in LACC without functional imaging generally achieve AUCs in the 0.65–0.75 range, whereas 18F-FDG PET/CT and MRI-based radiomics models report values of 0.80 or higher [38,39,40], and perioperative risk models in gynecological oncology surgery built on large multinational datasets achieve moderate discrimination even with many more events [14,15]. The present sample size of 25 events falls far below the number required by contemporary sample-size criteria for prediction models [34], and the Firth analysis confirmed that maximum-likelihood estimates were slightly inflated. We therefore explicitly refrain from presenting the model as a clinically meaningful prediction tool; it is an exploratory analysis whose value lies in nominating variables and in quantifying how much (or how little) they explain.

The strengths of the study are the consecutive, registry-based enrollment of a contemporary real-world cohort during the first year of INTERLACE-type induction chemotherapy at a Romanian tertiary center, the explicit separation of upfront, completion, and salvage surgery, a pre-specified and internally validated analysis with penalized and restricted sensitivity analyses, and transparent reporting of the model’s limited performance. The limitations are substantial and are listed in order of importance.

First, the outcome combines clinically distinct events. Planned upfront surgery is an initial treatment decision, completion surgery reflects residual disease and institutional management strategy, and salvage surgery is a subsequent oncological event; pooling them into a single binary endpoint limits the clinical meaning of the model. The sensitivity analysis restricted to post-treatment surgery, in which the directions of association were preserved, only partially addresses this, because it contained 11 events and could not be modeled multivariably. Second, the cohort is heterogeneous in diagnosis and treatment pathway. Although 89.9% of patients had cervical carcinoma and the cervical-only analysis reproduced the primary estimates, the inclusion of endometrial, vulvar, vaginal, and urogenital tumors, which have different indications for surgery, radiotherapy, and salvage, adds noise that a cohort of 69 patients cannot absorb, as illustrated by the ANOVA result for the ΔTD that was driven entirely by two urogenital cases. Third, the radiotherapy component is described at the level of prescription, technique, and brachytherapy utilization, but individual HR-CTV D90 values, organ-at-risk D2cc doses, OTT, and treatment interruptions were not extracted; without these, the relationship between radiotherapy quality, residual disease, and surgery cannot be fully interpreted. Moreover, the ΔTD itself is an exploratory institutional treatment-deviation metric: as an unsigned difference it assigns similar positive values to additional dose and to dose reduction or premature termination, and it should not be read as a conventional dosimetric predictor.

Fourth, follow-up is short and immature. Accrual ran from April 2024 to April 2025 with a data lock at the end of that period, giving a median follow-up of 11.2 months overall and only 8.6 months in the induction arm, where fewer than half of patients had reached 6 months after treatment. Because the median interval from the end of primary treatment to completion or salvage surgery in the remainder of the cohort was 4.1 months, with observed intervals extending to 9.6 months, a proportion of induction patients had not yet passed the point at which such a decision would be made. The finding that no induction patient required surgery is consequently a preliminary observation rather than a demonstrated outcome, and the surgical rate in that arm may rise with longer follow-up. In addition, the mechanism proposed to link rural residence to surgery was not measured: overall treatment time, travel distance, treatment interruptions, and adherence were not collected, so the pathway from geography to attrition to prolonged treatment to surgery remains untested. The Delta Target Dose was coded as 0 Gy for the 11 patients who received no radiotherapy, a convention that conflates absent irradiation with perfect dose delivery and that the pre-specified analysis restricted to irradiated patients (Section 3.5) only partially mitigates.

Fifth, the model lacked integration with advanced biological and functional radiological biomarkers. The literature heavily emphasizes 18F-FDG PET/CT, which offers superior diagnostic sensitivity and specificity for detecting residual disease compared to early MRI [39]. Integrating MRI-based radiomics drastically improves prognostic accuracy [38]. Incorporating functional imaging alongside genomic or HPV markers may improve the discrimination of future models [40]. For the present dataset, however, this is a secondary consideration: the more fundamental gaps are the heterogeneous population, the immature follow-up, the absence of oncological endpoints, and the missing standardized radiotherapy parameters listed above. Sixth, the cohort is overwhelmingly composed of cervical squamous carcinoma (85.5%) and of female patients (97.1%), with only two urogenital cases. The findings therefore apply to locally advanced cervical and other gynecological malignancies and should not be generalized to genitourinary cancers. Seventh, the sample of 25 events supports at most four covariates and no covariate reached statistical significance, so that the study is underpowered to confirm or exclude moderate associations; the analysis is exploratory and no adjustment for multiplicity was applied.

The present findings define the design of the confirmatory study rather than a clinical algorithm. That study should (1) enroll a homogeneous population of LACC (FIGO IIB–IVA) treated with comparable definitive intent, excluding upfront surgical candidates and non-cervical diagnoses; (2) ensure mature follow-up of at least 2 years and use clinically meaningful oncological endpoints, namely treatment response at 3 months, local control, recurrence, and progression-free survival, with completion and salvage surgery analyzed as separate, clearly defined events; (3) record standardized radiotherapy and brachytherapy parameters (EQD2 HR-CTV D90 and D98, D2cc of bladder, rectum, sigmoid and bowel, OTT, interruptions, and brachytherapy completion) according to EMBRACE II [31]; (4) measure the access-related variables needed to test the geographic hypothesis (travel distance and time, interruptions, adherence); (5) record chemotherapy cycles by phase and relative to the surgical decision; and (6) follow the sample-size guidance for prediction models [34], which for an outcome frequency of about 20% and 6–8 candidate variables implies several hundred patients, so that a multi-institutional design will be needed. These six elements, namely a homogeneous LACC population, mature follow-up, clinically meaningful oncological endpoints, standardized radiotherapy parameters (HR-CTV dose, organ-at-risk doses, OTT, interruptions, and brachytherapy completion), access-related variables, and adequate sample size, are the fundamental priorities for this dataset. Only as additional, longer-term extensions, once such a cohort is available, could 18F-FDG PET/CT response, MRI-based radiomics, and HPV or genomic markers be integrated [38,39,40,41] and radiosensitization strategies such as hyperthermia be evaluated in the same framework [42]. To address the short follow-up, the institutional registry from which this cohort was drawn remains open: all 69 patients are being followed with 3-monthly clinical and 6-monthly imaging assessments, a second data lock is scheduled at 24 months after the last enrollment (April 2027), and the surgical status of the induction group will be reported with the corresponding 2-year local control and progression-free survival. Prospective collection of OTT, travel distance, and interruption data has been incorporated into the registry for patients enrolled from 2025 onward.

## 5. Conclusions

In this single-center observational cohort of predominantly cervical squamous carcinoma, no patient treated with dose-dense induction chemotherapy required surgery during the available follow-up. Because allocation was not randomized, was confounded by diagnosis and stage, and was observed over a median of only 8.6 months, this finding is hypothesis-generating and cannot establish that induction chemotherapy removes the need for surgical salvage; in a pathway in which chemoradiotherapy is itself the definitive treatment, the absence of surgery is not a surrogate of efficacy. Rural provenience was associated with higher odds of surgery, but the association did not reach statistical significance (OR 0.343 for urban residence, 95% CI 0.113–1.039, p = 0.059), and the logistical mechanism commonly invoked to explain it was not measured in this study.

The multivariable model incorporating provenience, age, the Delta Target Dose, and systemic treatment intensity showed fair apparent discrimination (AUC 0.717), which fell to 0.672 after bootstrap optimism correction, with a calibration slope of 0.781 and an overall accuracy of 73.91% against a no-information rate of 63.8%. Its sensitivity of 0.480 means it misses slightly more than half of the patients who ultimately require surgery. None of its four covariates reached statistical significance. The model is therefore an exploratory analysis and not a clinical prediction tool; its direction of association was stable across penalized and restricted sensitivity analyses, but its endpoint pools clinically distinct surgical events and its cohort is heterogeneous. Its value lies in formulating hypotheses about candidate variables, in particular geographic provenience and planning-to-delivery treatment deviation (the exploratory ΔTD metric), that merit prospective evaluation in larger, multi-institutional cohorts with mature follow-up, standardized measurement of overall treatment time and access-related factors and, as a further extension, integration of functional imaging and biomarkers.

## Figures and Tables

**Table 1:** Baseline characteristics of the cohort by treatment group.

Characteristic	All (*n* = 69)	Group A, Induction (*n* = 27)	Group B, Classic (*n* = 7)	Group C, No Induction (*n* = 35)	*p*
Age, years, mean ± SD	54.3 ± 11.2	52.1 ± 9.8	55.4 ± 10.6	55.8 ± 12.3	0.42
Female sex, *n* (%)	67/69 (97.1)	27/27 (100)	7/7 (100)	33/35 (94.3)	0.62
Rural provenience, *n* (%)	31/69 (44.9)	12/27 (44.4)	3/7 (42.9)	16/35 (45.7)	1.00
Primary diagnosis, *n* (%)					0.008
Cervical squamous carcinoma	59/69 (85.5)	26/27 (96.3)	6/7 (85.7)	27/35 (77.1)	
Cervical adenocarcinoma	3/69 (4.3)	1/27 (3.7)	0/7 (0)	2/35 (5.7)	
Endometrial carcinoma	2/69 (2.9)	0/27 (0)	0/7 (0)	2/35 (5.7)	
Vulvar carcinoma	2/69 (2.9)	0/27 (0)	0/7 (0)	2/35 (5.7)	
Vaginal carcinoma	1/69 (1.4)	0/27 (0)	1/7 (14.3)	0/35 (0)	
Urogenital carcinoma	2/69 (2.9)	0/27 (0)	0/7 (0)	2/35 (5.7)	
FIGO stage, cervical cases (*n* = 62), *n* (%)					<0.001
IB3–IIA	12/62 (19.4)	0/27 (0)	0/6 (0)	12/29 (41.4)	
IIB	26/62 (41.9)	13/27 (48.1)	3/6 (50.0)	10/29 (34.5)	
IIIA–IIIC	20/62 (32.3)	11/27 (40.7)	3/6 (50.0)	6/29 (20.7)	
IVA	4/62 (6.5)	3/27 (11.1)	0/6 (0)	1/29 (3.4)	
ECOG performance status 0–1, *n* (%)	61/69 (88.4)	25/27 (92.6)	6/7 (85.7)	30/35 (85.7)	0.71
Radiotherapy received, *n* (%)	58/69 (84.1)	27/27 (100)	7/7 (100)	24/35 (68.6)	0.001
Delta Target Dose, Gy, median (IQR)	0.9 (0–3.6)	1.2 (0–3.4)	2.0 (0.6–4.8)	0.6 (0–3.9)	0.47
Chemotherapy cycles ^1^, mean ± SD	4.6 ± 1.9	4.8 ± 0.9	4.7 ± 1.6	4.4 ± 2.5	0.73
Follow-up, months, median (IQR)	11.2 (7.4–14.9)	8.6 (5.9–10.8)	12.4 (9.1–15.2)	13.0 (9.8–16.1)	<0.001

^1^ Platinum-containing cycles delivered concurrently with radiotherapy, as adjuvant treatment, or subsequently, excluding induction cycles. Categorical variables are given as *n*/*N* (%), where *N* is the number of patients in the column or, for the FIGO stage rows, the number of cervical carcinoma cases in the column (62 overall; 27, 6, and 29 in Groups A, B, and C). *p* values from ANOVA, Kruskal–Wallis, or Fisher’s exact test. ECOG, Eastern Cooperative Oncology Group; FIGO, International Federation of Gynecology and Obstetrics; IQR, interquartile range; SD, standard deviation.

**Table 2:** Radiotherapy and brachytherapy characteristics of irradiated patients (*n* = 58).

Parameter	All Irradiated (*n* = 58)	Group A (*n* = 27)	Group B (*n* = 7)	Group C (*n* = 24)
Treatment intent, definitive/adjuvant, *n*	53/5	27/0	7/0	19/5
EBRT prescription, *n* (%)				
45 Gy/25 fractions	41 (70.7)	20 (74.1)	5 (71.4)	16 (66.7)
50.4 Gy/28 fractions	17 (29.3)	7 (25.9)	2 (28.6)	8 (33.3)
Nodal SIB 55–57.5 Gy, *n* (%)	19 (32.8)	10 (37.0)	3 (42.9)	6 (25.0)
Technique VMAT, *n* (%)	58 (100)	27 (100)	7 (100)	24 (100)
Concurrent weekly cisplatin, definitive cases, *n* (%)	49/53 (92.5)	26 (96.3)	6 (85.7)	17 (89.5)
≥5 concurrent cycles, *n* (%)	44/53 (83.0)	23 (85.2)	5 (71.4)	16 (84.2)
Carboplatin substitution, *n*	4	1	1	2
Brachytherapy delivered, definitive cases, *n* (%)	41/53 (77.4)	22 (81.5)	5 (71.4)	14 (73.7)
HDR 4 × 7 Gy completed, *n* (% of started)	36 (87.8)	20 (90.9)	4 (80.0)	12 (85.7)
Interstitial component, *n*	9	6	1	2
Not delivered: EBRT boost/declined/progression, *n*	5/4/3	2/2/1	1/0/1	2/2/1
Planning aim HR-CTV D90 EQD2 ≥ 85 Gy ^2^	yes	yes	yes	yes
Delta Target Dose, Gy, median (IQR)	1.8 (0–4.2)	1.2 (0–3.4)	2.0 (0.6–4.8)	2.4 (0–5.1)
≥5 Gy, *n* (%)	12 (20.7)	4 (14.8)	2 (28.6)	6 (25.0)

^2^ Departmental planning aim; individual HR-CTV D90, organ-at-risk D2cc, overall treatment time, and interruptions were not available in the study database. EBRT, external beam radiotherapy; EQD2, equivalent dose in 2 Gy fractions; HDR, high dose rate; HR-CTV, high-risk clinical target volume; IQR, interquartile range; SIB, simultaneous integrated boost; VMAT, volumetric modulated arc therapy.

**Table 3:** Oncological surgery by indication, procedure, and treatment group.

Surgery	All (*n* = 69)	Group A (*n* = 27)	Group B (*n* = 7)	Group C (*n* = 35)
Any oncological surgery, *n* (%)	25/69 (36.2)	0/27 (0)	2/7 (28.6)	23/35 (65.7)
Planned upfront surgery, *n*	14	0	0	14
Radical hysterectomy + pelvic lymphadenectomy	8	0	0	8
Total hysterectomy + BSO (endometrial)	2	0	0	2
Radical vulvectomy	2	0	0	2
Radical cystectomy/partial penectomy	1/1	0	0	1/1
Completion surgery for residual disease, *n*	8	0	2	6
Extrafascial hysterectomy	5	0	1	4
Radical hysterectomy	3	0	1	2
Salvage surgery for recurrence, *n*	3	0	0	3
Pelvic exenteration	2	0	0	2
Radical hysterectomy	1	0	0	1
Post-treatment surgery (completion + salvage), *n* (%) ^3^	11/55 (20.0)	0/27 (0)	2/7 (28.6)	9/21 (42.9)
Interval from end of treatment to post-treatment surgery, months, median (range)	4.1 (1.8–9.6)	–	3.6 (2.9–4.3)	4.3 (1.8–9.6)

^3^ Denominator excludes the 14 patients with planned upfront surgery. BSO, bilateral salpingo-oophorectomy.

**Table 4:** Exploratory multivariable logistic regression for any oncological surgery: primary model and sensitivity analyses.

Covariate	Primary Model (*n* = 69, 25 Events) OR (95% CI); *p*	Firth Penalized (*n* = 69) OR (95% CI); *p*	Irradiated Only (*n* = 58, 16 Events) OR (95% CI); *p*	Cervical Only (*N* = 62, 19 Events) OR (95% CI); *p*
Urban vs. rural provenience	0.343 (0.113–1.039); 0.059	0.372 (0.128–1.041); 0.061	0.31 (0.09–1.08); 0.066	0.35 (0.11–1.12); 0.078
Age, per year	1.012 (0.966–1.060); 0.61	1.011 (0.967–1.057); 0.63	1.02 (0.97–1.07); 0.49	1.01 (0.96–1.06); 0.71
Delta Target Dose, per Gy	1.109 (0.990–1.244); 0.076	1.098 (0.986–1.226); 0.089	1.097 (0.968–1.243); 0.15	1.10 (0.98–1.24); 0.11
Chemotherapy cycles, per cycle	1.298 (0.973–1.732); 0.076	1.271 (0.968–1.677); 0.083	1.24 (0.90–1.71); 0.19	1.30 (0.95–1.79); 0.10
Apparent AUC (95% CI)	0.717 (0.592–0.842)	0.714 (0.588–0.840)	0.708 (0.556–0.860)	0.712 (0.580–0.844)
Optimism-corrected AUC	0.672	0.675	0.641	0.658
Calibration slope (bootstrap)	0.781	0.842	0.702	0.749
Accuracy/NIR	73.9%/63.8%	73.9%/63.8%	75.9%/72.4%	72.6%/69.4%
Sensitivity/specificity	0.480/0.886	0.480/0.886	0.375/0.905	0.421/0.860

AUC, area under the receiver operating characteristic curve; CI, confidence interval; NIR, no-information rate; OR, odds ratio. Treatment group could not be included because of perfect separation (no events in Group A).

**Table 5:** Classification table of the primary exploratory logistic regression model at a predicted probability threshold of 0.5 (*n* = 69).

Model Prediction (Threshold 0.5)	Surgery Performed (*n* = 25)	No Surgery (*n* = 44)	Total
Predicted surgery (*p* ≥ 0.5)	12 (true positive)	5 (false positive)	17
Predicted no surgery (*p* < 0.5)	13 (false negative)	39 (true negative)	52
Total	25	44	69

Accuracy 73.9% (51/69; 95% CI 61.9–83.7) versus a no-information rate of 63.8% (exact binomial *p* = 0.049); sensitivity 0.480 (12/25); specificity 0.886 (39/44); positive predictive value 0.706 (12/17); negative predictive value 0.750 (39/52); Cohen’s κ 0.39.

## Data Availability

The raw data supporting the conclusions of this article will be made available by the authors on request.

## Abbreviations

The following abbreviations are used in this manuscript:

18F-FDG PET/CT	18F-fluorodeoxyglucose Positron Emission Tomography/Computed Tomography
AI	Artificial Intelligence
ANOVA	Analysis of Variance
AUC	Area Under the Curve
AUROC	Area Under the Receiver Operating Characteristic
BSO	Bilateral salpingo-oophorectomy
CC BY	Creative Commons Attribution
CCRT	Concurrent platinum-based chemoradiotherapy
CI	Confidence Interval
CTI	Chemotherapy treatment intensity
D2cc	Minimum dose to the most exposed 2 cm^3^ of an organ at risk
D90	Dose covering 90% of the target volume
df	Degrees of freedom (used in statistical tables)
EBRT	External Beam Radiotherapy
ECOG	Eastern Cooperative Oncology Group
EPV	Events Per Variable
EQD2	Equivalent Dose in 2 Gy fractions
FIGO	International Federation of Gynecology and Obstetrics (staging system)
Gy	Gray (unit of radiation dose)
HDR	High Dose Rate (brachytherapy)
HPV	Human Papillomavirus
HR-CTV	High-risk clinical target volumes
IGABT	Image-Guided Adaptive Brachytherapy
IQR	Interquartile Range
LACC	Locally advanced cervical cancer
LCP	Local Control Probability
MRI	Magnetic Resonance Imaging
NACT	Neoadjuvant chemotherapy
NIR	No-Information Rate
NPV	Negative Predictive Value
OR	Odds Ratio
OTT	Overall treatment time
PPV	Positive Predictive Value
RECIST	Response Evaluation Criteria in Solid Tumors
ROC	Receiver Operating Characteristic
SD	Standard Deviation
SIB	Simultaneous integrated boost
TNM	Tumor, Node, Metastasis (oncological staging system)
TRIPOD	Transparent Reporting of a multivariable prediction model for Individual Prognosis Or Diagnosis
VIF	Variance Inflation Factors
VMAT	Volumetric modulated arc therapy
ΔTD	Delta Target Dose
